# Biodegradability and Ecotoxicity Profiles of Choline Acetate, Betaine, and L-Proline NADESs: A Hidden Threat for Eutrophication?

**DOI:** 10.3390/molecules31020262

**Published:** 2026-01-12

**Authors:** Nandish M. Nagappa, Angelica Mero, Elena Husanu, Zeba Usmani, Matteo Oliva, Matilde Vieira Sanches, Giorgia Fumagalli, Andrea Mele, Andrea Mezzetta, Nicholas Gathergood, Lorenzo Guazzelli, Carlo Pretti, Yevgen Karpichev

**Affiliations:** 1 Department of Chemistry and Biotechnology, Tallinn University of Technology (TalTech), 12618 Tallinn, Estonia; nandish.mudegowdru@taltech.ee (N.M.N.);; 2 Department of Pharmacy, University of Pisa, 56126 Pisa, Italy; 3 Center for Material Interfaces, Istituto Italiano di Tecnologia, 56025 Pontedera, Italy; 4 Interuniversity Consortium of Marine Biology and Applied Ecology “G. Bacci”, 47128 Livorno, Italy; 5 Department of Biology & Centre for Environmental and Marine Studies (CESAM), University of Aveiro, 3810-193 Aveiro, Portugal; 6 Dipartimento di Chimica, Materiali e Ingegneria Chimica “Giulio Natta”, Politecnico di Milano, 20133 Milano, Italy; 7 School of Chemistry, College of Science, University of Lincoln, Lincoln LN6 7TS, UK; 8 Department of Veterinary Sciences, University of Pisa, 56122 Pisa, Italy

**Keywords:** naturally available deep eutectic solvents, biodegradation, ecotoxicity, *Raphidocelis subcapitata*, closed bottle test, hydrogen bond donor, hydrogen bond acceptor

## Abstract

Deep Eutectic Solvents (DESs), particularly Naturally Available Deep Eutectic Solvents (NADESs), are increasingly regarded as green solvents due to their low vapor pressure, non-flammability, thermal stability, strong solvent power, and low toxicity. In line with Green Chemistry principles, the use of renewable and biocompatible components such as amino acids, lipids, and naturally derived acids enables the development of more sustainable solvent systems. This study addresses the need for environmentally safer NADESs by evaluating their physico-chemical suitability and environmental impact. Fifteen NADESs were prepared using naturally derived components and assessed for environmental safety. Biodegradability was evaluated using the OECD 301D Closed Bottle Test (CBT), while toxicity toward *Raphidocelis subcapitata* was examined to characterize ecotoxicological behavior. The results demonstrated that the synthesized NADESs exhibit high biodegradability levels and low toxicity toward microalgae. Toxicity control indicated no significant inhibition of microbial activity during biodegradation, suggesting favorable environmental compatibility. Overall, the findings indicate that the NADESs represent more sustainable solvent alternatives with low toxicological profiles. However, the potential role of these compounds in enhancing eutrophication processes cannot be excluded and warrants further investigation.

## 1. Introduction

In the advent of the sustainability and green chemistry era, there is an ongoing quest to discover safer and more environmentally friendly solvents to replace traditional ones derived from fossil-derived chemicals [1]. These conventional solvents pose significant environmental threats during production, use, and disposal [2]. The end-of-life cycle for these solvents is a problem because they are not biodegradable. This means that they must be burned, which creates CO_2_ emissions, as they are often non-biodegradable. In addition, the impact of these solvents on human health has recently become a cause for concern in various industrial sectors. For example, *N,N*-dimethyl formamide (DMF) has recently been highlighted for its reproductive toxicity [3]. Consequently, its use, particularly in the pharmaceutical industry, has been prohibited within the European Union. In this context, the investigation of sustainable alternatives to fossil-based solvents has intensified in recent years. Among the neoteric solvents, the Deep Eutectic Solvents (DESs) represent a versatile alternative to volatile organic solvents, including those of natural origin [4]. The recent popularity of DESs is mainly due to their simple preparation and their relatively low cost [5,6]. DESs are typically obtained by mixing two or more compounds [7], which determines a decrease in the melting point of the mixture below that expected for the ideal eutectic [8]. At the basis of this deviation, there is the strong interaction between a hydrogen bond donor (HBD) and a hydrogen bond acceptor (HBA) that creates a strong microheterogeneity at the molecular level [9]. However, the real key to the success of DES is their tuneability, which allows for the customization of the system to suit specific needs. Simply modifying the chemical composition of the mixture allows its chemical and physical properties to be adapted for a specific application. An additional degree of modularity can be achieved by adjusting the molar ratio between the components [10]. A subset of DESs, inspired by nature and derived from plant-based products and other bio-resources, are called Natural Deep Eutectic Solvents (NADESs) [11]. In fact, they are composed entirely of bio-based compounds, which is the reason behind their biocompatibility [12,13,14].

Although the definition of DESs does not have a direct implication on most features, initially, the green characteristics of DESs were assumed based on the pharmaceutically acceptable profile that was reported in the material safety data sheets of the selected individual components [15]. Later, a few studies were devoted to investigating the DESs toxicity profile [16]. Indeed, bio-based chemicals may not be completely biodegradable, and hence, are not 100% environmentally friendly. However, despite the great interest of academia and industry in DESs and NADESs applications for gas capture [17], biomass valorization [18,19], wastewater entrapment [20] and wastewater treatment [21], material synthesis [22], the toxicity of these compounds is still an underrated issue. Limited recent research studies have investigated NADESs toxicity on different targets, such as human cells [23,24,25], bacteria [25,26,27,28], yeasts [25], invertebrates and microalgae [12,26,27], and fish [29], sometimes reporting conflicting results. Indeed, what emerged from different papers was a pool of different behaviors of HBA/HBD if assessed singularly or, instead, in a NADESs composition [12]. Moreover, several studies support the idea of DESs as “eco-friendly”, assuming they are not toxic, given the fact they are formed by naturally occurring ingredients and are, for this reason, bio-renewable, biodegradable and bio-assimilable [30,31], while other publications report a clear toxicity linkable to some DESs components [23]. 

Among the NADESs components, choline is the most used compound to prepare DESs [32], due to its high biodegradability [33] and low toxicity [31] as well as low cost and widespread availability [34]. Although found in living organisms, the choline cation currently on the market is of fossil origin and is obtained from trimethylamine and ethylene oxide [33]. In particular, choline acetate (ChA) and bitartrate (TA) are much less commonly used than choline chloride (ChCl) in the preparation of DES, but they are a valuable halogen-free alternative. For example, DES based on ChTA have recently been used to increase the solubilization of drugs [35], plasticize starch [36] and extract flavonoids [37]. ChA-based DESs have also recently been studied from a physico-chemical and application perspective [38,39,40,41]. For example, ChA:glycolic acid (GA), ChA:levulinic acid (LA) and ChA:imidazole (Im) showed excellent performance in hemicellulose solubilization and were used in Kraft cellulose purification [42]. On the other hand, betaine (trimethylglycine, Bet), a zwitterionic compound bearing both formal positive and negative charges, has also been used to prepare DESs [5] and has been even suggested as the universal HBA [43]; like choline, betaine is also biocompatible [44], has a low toxicity profile [45,46], and is readily biodegradable. However, in contrast to ChCl, Bet can be derived from renewable sources and, in particular, by the transformation of the by-product of sugar production [42]. The use of Bet-based DESs has grown significantly in recent years and they are being used in numerous applications [47,48,49,50].

The proteinogenic amino acid L-proline (L-Pro) has recently received attention for designing ILs [51,52,53] and NADESs [54,55] for different applications. The ability of L-Pro to form enamines or imines when reacting with carbonyl groups, as well as its ability to induce chirality promoted by the cyclic structure of the molecule, have been exploited in a wide range of organic synthetic methodologies. L-Pro can promote a very broad diversity of transformations, which can be explained by the multiple catalytic roles allowed by its structural features. 

Similarly, L-Pro can behave both as an acid or Brønsted base, or even show both behaviors during a mechanism, therefore, being a bifunctional catalyst. It is the only natural amino acid with a secondary amine functionality, a feature that raises the nitrogen p*K*_a_ and increases its nucleophilicity in comparison to other amino acids. L-Pro has several additional advantages, such as being inexpensive, commercially available, non-toxic and easily recoverable, which are important properties from the point of view of green chemistry [56]. L-Pro has recently become widely used as an ingredient in supplements, health foods and cosmetics. The use of L and D-Pro as an HBA in the preparation of DESs allows us to develop new reaction media with a dual solvent/catalyst role [57].

Previous research published by the co-authors of this study presented one of the most comprehensive ecotoxicological screens of NADESs evaluated using a broad battery of marine and freshwater bioassays [12]. The overall findings revealed a general absence of acute toxicity across the NADESs tested; however, some NADESs induced algal biostimulation responses in freshwater assays. This suggested that while NADESs are often labeled as “sustainable”, their ecological effects, particularly for primary producers, may be more complex than predicted by single-species toxicity tests alone, especially under conditions where biostimulation could influence ecosystem functioning. Those results highlighted the need for further investigation of both ecotoxicological endpoints and biodegradation behavior to more reliably assess the environmental safety of NADESs.

**Figure 1:**
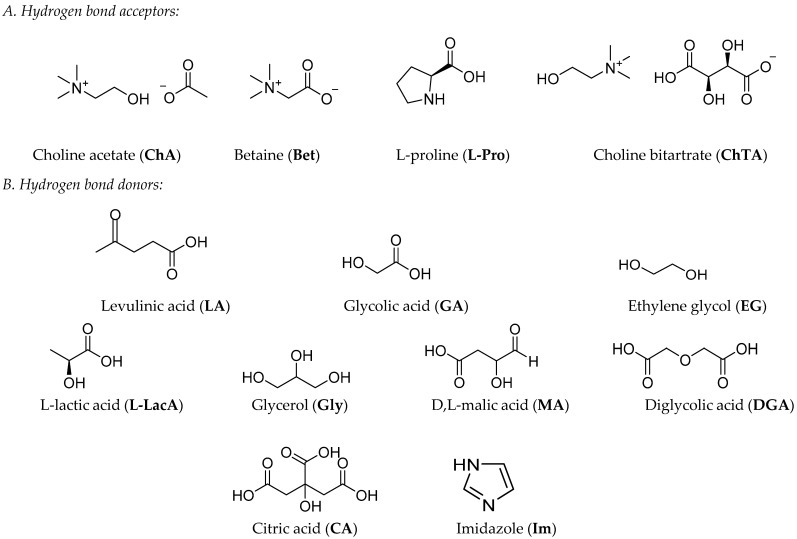
Chemical structures of hydrogen bond acceptors (**A**) and hydrogen bond donors (**B**) and their abbreviations used in this work.

In this study, a series of 15 different choline, betaine, and L-proline-based NADESs (Table A1) and their single components (Figure 1) was assessed for their biodegradability using the aerobic biodegradation method [58], in order to evaluate their persistence in aquatic systems and the potential release of nutrients, such as N and P by molecule dissolution.

Moreover, we evaluated the potential ecotoxic effect of these compounds and their respective single components in terms of freshwater microalgae *Raphidocelis subcapitata* growth stimulation and/or inhibition, hypothesizing an underrated effect of NADESs as potential eutrophication substances. 

## 2. Results

### 2.1. Biodegradation Results of NADESs 

The biodegradability tests conducted for all of the test substances for 28 days were proven to be valid as the control, readily biodegradable sodium acetate, was eliminated by at least 60% by the end of 14 days. 

**Figure 2:**
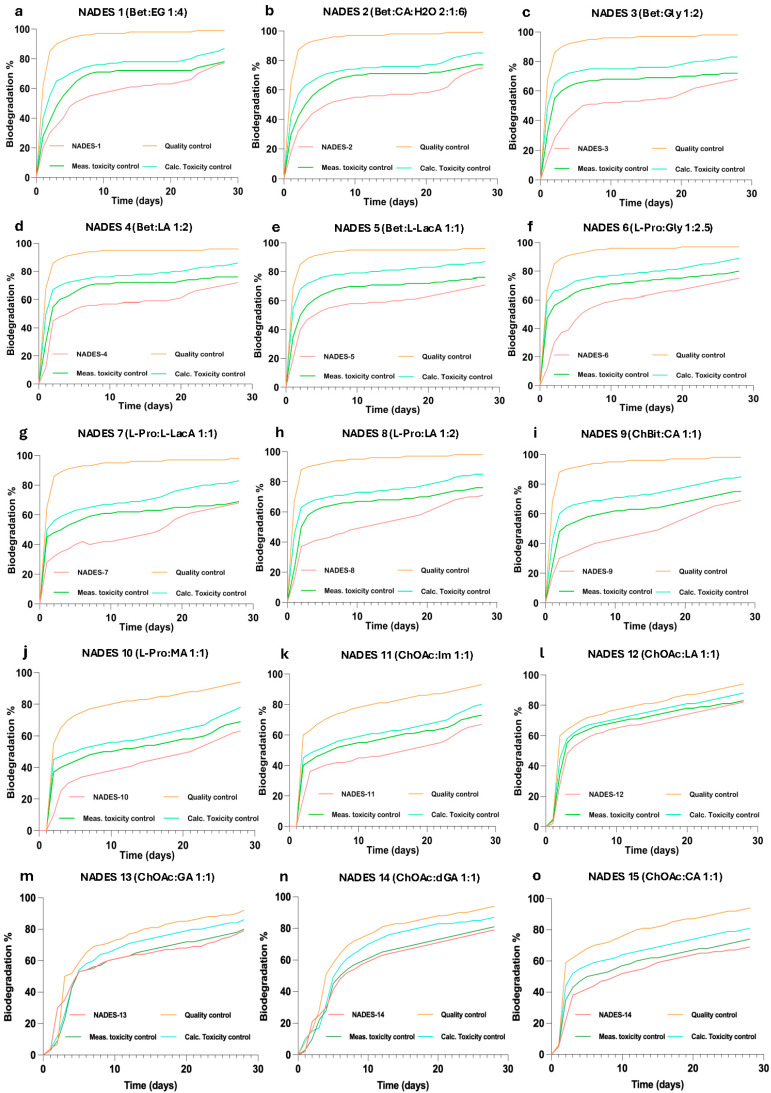
Biodegradability profiles of the Closed Bottle Test (CBT OECD 301D, 28 days) for the studied NADESs: (**a**) Bet:EG; (**b**) Bet:CA; (**c**) Bet:Gly; (**d**) Bet:LA; (**e**) Bet:L-LacA; (**f**) L-Pro:Gly; (**g**) L-Pro:L-LacA; (**h**) L-Pro:LA; (**i**) ChTA:CA; (**j**) L-Pro:MA; (**k**) CA:Im; (**l**) ChA:LA; (**m**) ChA:GA; (**n**) ChA:DGA; (**o**) ChA:CA. Quality control series: sodium acetate (6.41 mg/L) in inoculated mineral medium, used to verify microbial activity and test validity; rapid biodegradation of sodium acetate confirms inoculum viability. Measured toxicity control: contains both sodium acetate and the test compound in their respective concentrations, used to assess potential inhibitory or toxic effects of the test compound on microbial activity by comparison with the quality control series. Calculated toxicity control: theoretically calculated biodegradation curve from the sum of the expected oxygen consumption of the reference series and the test series, assuming no inhibitory effects of the test compound on microbial activity. Used to identify any potential inhibitory effects of test substance on microbial activity.

All the tested NADESs, as well their single components, were observed to be classified as “readily biodegradable”, showing biodegradation values > 60% after a 28-day Closed Bottle Test (Table A2). The only exception was Im, with a biodegradation value of 23% as an indisvidual HBD component, although when in combination with ChAc (NADES 11), it seemed to not affect whole NADESs’ biodegradability score (67%). The biodegradability profiles observed for the studied NADESs are presented in Figure 2a–o.

### 2.2. Raphidocelis Subcapitata Growth Bioassay 

**Figure 3:**
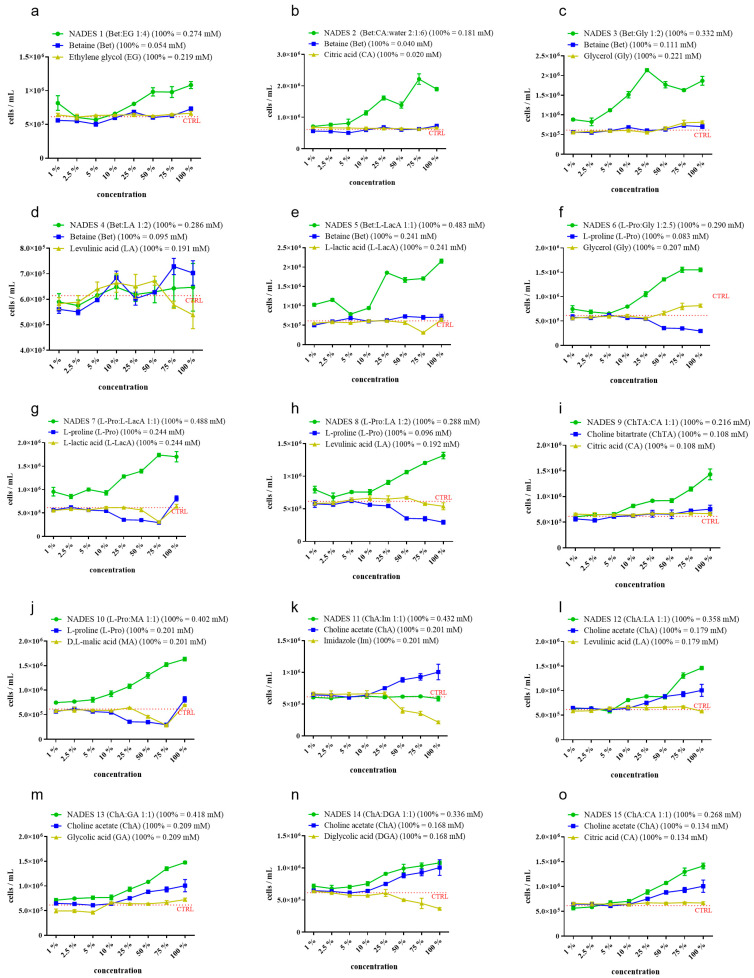
Concentration–response graphs of *R. subcapitata* exposed to NADESs and to their single components at the relative concentration as in the whole compound at the same concentration. The red dotted line is the mean algal concentration measured in the controls. (**a**) Bet:EG; (**b**) Bet:CA; (**c**) Bet:Gly; (**d**) Bet:LA; (**e**) Bet:L-LacA; (**f**) L-Pro:Gly; (**g**) L-Pro:L-LacA; (**h**) L-Pro:LA; (**i**) ChTA:CA; (**j**) L-Pro:MA; (**k**) CA:Im; (**l**) ChA:LA; (**m**) ChA:GA; (**n**) ChA:DGA; (**o**) ChA:CA. Results are expressed as mean algal concentration (cells mL^−1^) ± standard deviation (n = 3) for each tested concentration of each tested substance.

Figure 3 represents the growth behavior of *R. subcapitata* algae exposed to different concentrations of 15 NADESs. Each graphic shows the differences, in terms of concentration–response trend, between NADESs and their components. The concentration–response curve showed an algal growth stimulation with increasing concentrations in thirteen NADESs compared to that of the control. The only two tested NADESs where the algal growth was not different from that of the control were NADES 4 (Bet:LA) (Figure 3d) and NADES 11 (ChA:Im) (Figure 3k).

The concentration–response curve showed an algal growth stimulation with increasing concentrations in thirteen NADESs compared to the control. The only two tested NADESs where the algal growth was not different from that of the control were NADES 4 (Bet: LA, Figure 3d) and NADES 11 (ChA:Im, Figure 3k).

Among the NADESs components, the one with the most stimulating effect in terms of algal growth was choline acetate, which represents the HBA of NADESs 11–12–13–14–15 (Figure 3k–o). Following the same trend, L-Pro in NADESs 7 and NADES 10 (Figure 3g and Figure 3j, respectively) showed a slight growth stimulation at the maximum tested concentration, followed by a reduction in algal growth at 75%, 50% and 25%. The same compound, tested at concentrations as in NADESs 6 and 8 (Figure 3f and Figure 3h, respectively), showed an inhibiting effect until 50%. This is linked to the fact that the concentration of L-Pro in NADESs 6 and 8 at 50% was similar to L-Pro in NADESs 7 and 10 at 25%. A similar trend, as for L-Pro in NADESs 7 and 10, was observed for MA and L-LacA (Figure 3e,g,j). However, in these cases, the inhibition effect was limited to the concentrations of 50% and 75% for MA and only at 75% for L-LacA.

Bet and ChTA displayed a similar effect on algal growth, having both shown biostimulation at the 100% concentration of NADESs 1,2,3,5 and 9 (Figure 3a–c,e,i). In addition, a biomass increase was also found at 50 and 75% of Bet in NADES 5, and at 75% of ChTA in NADES 9. Contrastingly, Bet exhibited a different algal growth pattern in NADES 4 (Figure 3d), where three peaks of biostimulation were found at 10, 75 and 100%, and an inhibition peak was displayed at 2.5%.

Concerning other tested components, they showed no effect as their growth patterns were very similar to those of the respective controls.

ANOVA results, with Tukey’s post-test for multiple comparisons, for all concentration–response effects of each NADESs, are presented in Supporting Information (Table S1).

## 3. Discussion

Considering the continuously growing necessity of finding new green solutions aimed at decreasing the impact of human activities on the environment, the present study took in considering a pool of 15 NADESs, claimed as non-toxic systems, to evaluate their biodegradability potential and their effects on freshwater algal growth. The same analyses were accrued out on the single components to highlight possible differences when mixed in the NADESs. 

The current study represents the natural development of our previous research work on NADESs [12], where the pool of 15 NADESs was evaluated in terms of ecotoxicological effects on two batteries of tests, a marine test and a freshwater test. Due to interesting results obtained in terms of microalgal growth biostimulation, particularly emphasized in the freshwater environment, a deeper investigation on biodegradability and the single component effect, in respect to the formed DES system, was considered necessary. Observing, as an example, biodegradability percentages of NADES single components after 28 days, we may notice that Im was the one with the lowest biodegradation value (23%). We may suggest that the inoculum used was likely deficient in key breakdown pathways for this particular chemical, since the biodegradability rose to 67% in the NADES with choline acetate, underlining complex interactions between components in whole NADES formulation.

Despite the very limited toxic effect of NADESs observed with tests involving invertebrates, their potential impact on the environment, if improperly disposed of, may lie in their characteristic as a nutrient source, thus increasing the already large pool of substances with the potential to disrupt biogeochemical cycles at a global scale [59]. This disruption might be intensified by extreme natural events linked to a Global Change Scenario, such as thermal heatwaves [60] and ocean acidification [61]. Regarding the latter, how NADESs in aqueous solutions are able to reduce pH value and, as a consequence, enhanced algal growth was observed [12,62].

In addition to these statements, a ready biodegradability was observed for the majority of the assessed NADESs, with biodegradation values at 28 days between 60% and 81% for NADES compounds, and between 23% and 79% for their single components.

Our data coincides with the report on NADESs-based ChCl as HBA and a series of HBD (glycerol, ethylene glycol, urea, glucose, malonic acid, and lactic acid) studied by CBT [33], which demonstrated a biodegradability of beyond 60% in all cases. Irrespective of number of hydroxyl groups per mole or the presence of carboxylic derivatives, such as acids, esters, or amides, the biodegradability profiles were considered as “readily biodegradable”. This feature may lead the general opinion to consider NADESs as completely “green” and “eco-friendly” substitutes to conventional organic solvents. However, the fact that they are readily biodegradable may enhance the load of dissolved ions/molecules with nutritional value, such as choline, also known as vitamin B4, and its degradation products, emphasizing the occurrence of eutrophication phenomena [63].

Due to lack of literature treating NADES effects on microalgae, to better understand current observations, it is important to deeply analyze all obtained results and compare NADES effects with their single component behavior. Among the NADESs, those showing the highest induction of algal growth were NADES 2 (Bet:CA), 3 (Bet:Gly) and 5 (Bet:L-LacA). All of these three systems have betaine in their formulation, which, notwithstanding a lack of effect while tested as single component, strongly enhanced algal proliferation when combined with Gly, CA, or L-LacA. Moreover, NADESs containing L-Pro as HBA, which tend to slightly reduce algal load while being assessed as single component at high concentrations (>0.29 mM), displayed a growth-stimulating effect when in combination with other HBDs (NADESs 6–7–8–10). Differently from those cases, effects of NADESs with ChA (NADESs 11–12–13–14–15), appeared to be more influenced by this latter component with respect to all other HBAs, given the similar shape of concentration–effect curves. 

Moving the focus specifically to NADES components, it is possible to divide them into three main groups, depending on their effect. The first group, which comprises the majority of assessed compounds (betaine, ethylene glycol, citric acid, glycerol, glycolic acid, choline bitartrate, L-lactic acid, malic acid and levulinic acid), is composed by substances without significant effects, neither in enhancing nor inhibiting algal growth. The second group is composed of Im, L-Pro and DGA. These three compounds showed a limited inhibiting effect on algal growth when tested at concentrations > 0.08 mM for L-Pro, >0.107 mM for Im, and >0.1 mM for DGA (concentrations in the relative NADES solution, assessed at dilutions between 25% and 100%). The last group is composed of ChA only, which is present in NADESs 11–15 formulations (Figure 3k–o). As stated before, this particular compound showed a clear algal growth-enhancing effect, which followed the same concentration–response curve of the relative NADESs, appearing to be the main factor responsible for algal growth, if compared with concentration–response curves of each assessed whole NADESs. However, if compared with the relative whole system, the algal biomass measured at each ChA assessed concentration resulted in lower values. This, again, led us to hypothesize a synergistic effect between ChA and the other DES component, like in other assessed NADESs. The only exception was represented by NADES 11, where the two opposite effects of Im and ChA seemed to interact by simple addition (Figure 3k). 

This hypothetical synergistic effect of the two components in the NADES composition, observed as an emphasized “overgrowth” effect of the entire systems if compared with both single compounds until 25% as a threshold concentration, has already been observed for ChCl-based DESs [64,65], but also for other toxic DESs, such as glyceline, ethaline and reline [27]. This last result was also observed in the present work with NADES 11 (ChA:Im 1:1), which, as reported before, has Im in its formulation. Im is also known to be linked to aquatic toxicity in other kinds of NADES-related compounds, such as ionic liquids [12]. However, while Im showed a certain degree of algal growth inhibition, no relevant effects were observed when it was assessed in combination with ChA as a whole DES. 

Regarding biodegradability, with the exception of the previously discussed imidazole, no other relevant differences in terms of biodegradability percentages were observed between single components and NADESs, all with values still over 60%. However, the readiness of biodegradability of these compounds, considering that several of them, such as L-proline, betaine and choline acetate, contain nitrogen in their formulation, and the synergistic effect between some components in enhancing microalgal growth, may act as a perfect fertilizer with a potential eutrophication effect [66]. 

## 4. Materials and Methods

The NADESs used in this study and their composition, along with the ratio of the mixtures and their chemical structure, as well as the NADESs components, are listed in Table A1. 

### 4.1. Chemicals 

Choline acetate (ChA) 98% was purchased from IOLITEC (Heilbronn, Germany). Betaine (Bet) > 99% and L-Proline (L-Pro) > 99% were purchased from Tokyo Chemical Industry (TCI) (Tokyo, Japan). Ethylene glycol 99% (EG), levulinic acid (LA) 98%, imidazole (Im) 99%, and cholinium bitartarate (ChTA) > 98% were purchased from Thermo Fisher (Waltham, MA, USA). Glycerol (Gly) 99%, glycolic acid (GA) 99%, malic acid (MA) > 99%, diglycolic acid (DGA) 98%, and L-lactic acid (L-LacA) ≥ 98% were obtained from Sigma-Aldrich (Merck, Darmstadt, Germany). Ultrapure deionized water (Milli-Q Direct Water Purification System) was used for all solutions and media preparation. Potassium dichromate, K_2_Cr_2_O_7_ (1 g/L) was purchased as a dehydrated salt (ACS reagent grade, purity ≥ 99.0%) from Sigma-Aldrich and used as a reference toxicant for algal growth inhibition assay. The composition and molar ratios of NADESs tested in this study are reported in Table A1. NADESs were prepared following the methodologies described in the literature [16,42].

### 4.2. Biodegradability Assessment

In the assessment of the biodegradability of organic compounds, the initial and simple test used is the Closed Bottle Test (modified OECD 301D), usually referred to as the aerobic biodegradation test [67,68].

Aerobic biodegradation testing was performed using the modified Closed Bottle Test (CBT), based on OECD 301D guidelines [68,69]. CBT setup with modifications, where biological oxygen consumption is measured with an optode oxygen sensor system using PTFE-lined PSt3 oxygen sensor spots (Fibox 3 PreSens, Regensburg, Germany), allows the measurement of BOD without dispensing it from the stock solution each time for each test and thereby reducing the number of parallels, as once we open a bottle to measure its BOD, we cannot use it anymore because it is exposed to the atmosphere; so, in that case, we would keep a stock solution and pour it into a new measuring flask each time. The modified setup has also been shown to improve the reproducibility compared to the original OECD 301D guidelines [69,70]. Compared to other standard aerobic biodegradation tests, CBT is better suited for testing compounds with different physico-chemical properties. It is also one of most stringent OECD tests for biodegradability, as the amount of inoculum added is very low and, thus, compounds passing CBT 301D should show good biodegradation not only under artificial wastewater treatment conditions but also in soil and groundwater systems.

Experimental Setup. Each CBT run consisted of four different series, each of which was run in duplicates. First was quality control (reference series), in which readily biodegradable sodium acetate in a known concentration (6.41 mg/L) was added to a flask of mineral medium inoculated with effluent from a wastewater treatment plant. As sodium acetate is known to be rapidly biodegradable, it acted as a reference and control for monitoring the activity of microbes in the inoculum. In the test series, a studied compound as the sole source of carbon was added to the inoculated mineral medium. The test compound was added in a concentration corresponding to theoretical oxygen demand (ThOD) of approximately 5 mg/L. ThOD was calculated assuming nitrification would take place as each of the 25 studied compounds included nitrogen atom(s) in their structure. The toxicity control series, containing both sodium acetate and the test compound in their respective concentrations, were used to evaluate test the compounds’ toxicity against inoculum—if biodegradation values in these bottles were significantly lower compared to reference series, it was concluded that the test compound could be inhibiting or even being toxic to microbes in the WWTP effluent. To negate the effect of inoculum itself, blank bottles containing only inoculum and a mineral medium were added to each CBT run and the value of these bottles was subtracted from all the other bottles. To make sure seasonal variations in inoculum composition did not have an effect on biodegradation results, a total of 6 CBT runs from June to November were performed. 

Inoculum. Effluent from a wastewater treatment plant was collected from a municipal wastewater treatment plant in Tallinn, Estonia (Paljassaare wastewater treatment plant, 59°27′55.5″ N 24°42′08.8″ E). WWTP effluent was filtered through a cellulose filter (membrane ø 240 mm) before being used as inoculum for aerobic biodegradation testing. 

Results from each run were accepted if the following criteria were met: (i) difference in extremes of replicate values at the plateau is less than 20%, (ii) oxygen concentration in test series bottles must not fall below 0.5 mg/L at any time, (iii) sodium acetate in reference series must be degraded ≥ 60% by day 14. The blank bottles’ oxygen consumption was also monitored to avoid the possibility of the system turning from aerobic to anaerobic. In the CBT runs, the oxygen consumption in all of the blank bottles did not exceed 34% of the initial oxygen concentration.

### 4.3. Ecotoxicity Assessment

Stock and working solutions

3N-BBM+V (Bold Basal Medium with 3-fold nitrogen and vitamins), as the algal culture medium, was prepared according to CCAP (Culture Collection of Algae and Protozoa) guidelines. Medium pH value was corrected to 8.00 ± 0.1.

### 4.4. Naturally Available Deep Eutectic Solvents (NADESs) 

For all NADESs, a set of working concentrations was prepared, starting from 100 mg/L, which represents our 100%. Selected dilutions for each compound were 100–75–50–25–10–5–2.5–1%. Dilutions were carried out with algal culture medium 3N-BBM + V.

### 4.5. HBAs and HBDs 

For each HBA and HBD, 1 g/L stock solution was prepared in ultrapure water. These stock solutions were further diluted to test the concentration of each individual component, matching the concentration they had in the corresponding NADES mixture. Dilutions were carried out with algal culture medium 3N-BBM + V. Results were plotted as percentages indicating the relative amount of compound at that tested NADES concentration. 

### 4.6. Microalgal Bioassay 

*R. subcapitata* was purchased from the reference center CCAP (Culture Collection of Algae and Protozoa—Scottish Association for Marine Science/SAMS Research Services Ltd., Oban, Scotland, UK). Axenic cultures were kept in 100 mL glass flask stored at 20 ± 2 °C, under natural white illumination (6000–8000 lx) with a 16:8 dark/light photoperiod. 3N-BBM + V was used for culturing *R. subcapitata*. Cultures were renewed every two weeks. The growth assessment of the freshwater alga *R. subcapitata* (batch: CCAP 1052/1A) was performed following ASTM procedures [71].

Before the test started, an algal working batch was prepared by inoculating 2 mL of maintenance cultures in 20 mL of fresh medium, maintaining it at 20 ± 2 °C under continuous illumination (6000–8000 lx) in order to obtain a logarithmic-phase algal culture. After 72 h, the algal concentration in the working batch was measured and diluted to reach a concentration of 10^6^ cell/mL. For the growth inhibition bioassay, all samples at all concentrations were prepared in triplicate in sterile 24-well plastic plates. 20 μL of the diluted algal working batch was inoculated in each 2 mL replica of all samples and negative controls (medium). Plates were incubated at 20 ± 2 °C under continuous illumination (6000–8000 lx) for 72 h.

After 72 h, absorbance (λ = 670 nm) was measured in each well with a spectrophotometer (Jenway Genova Plus, Antylia Scientific, Chicago, IL, USA), using 1 cm optic-path plastic cuvettes. Algal concentration (Cells/mL) was calculated using the following equation, previously obtained by the CIBM (Livorno, Italy) research group:

$$Cells/mL=\frac{{Abs}_{670}}{{8\times 10}^{-8}}$$

The reference toxicant was potassium dichromate. Stock solution was prepared by dissolving the dehydrated salt in ultrapure water at a concentration of 1 g/L. From the stock solution, five different concentrations of potassium dichromate were prepared, respectively, directly in algal growth medium (1.8–1–0.56–0.32–0.18) mg/L to check the reliability of the test. The results obtained for this assay fell into the laboratory control chart (EC_50_ 0.742 mg/L (0.648–0.808). 

### 4.7. Statistical Analysis 

For statistical analysis, two-way ANOVA was performed, followed by Tukey’s multicomparison test to evaluate the difference in algae growth between (1) DESs and control (2) HBA/HBD and control (3) DESs and HBA/HBD (4) HBAs and HBDs. Statistical analysis was performed with Graph-Pad Prism 7 software (GraphPad Software, La Jolla, CA, USA, www.graphpad.com, accessed on 30 April 2025). Statistically significant differences were reported with asterisks: *p* < 0.05 (*), *p* < 0.01 (**), *p* < 0.001 (***). 

## 5. Conclusions

All the studied NADESs and the absolute majority of their components showed good biodegradation values (i.e., >60% = readily biodegradable). However, although they could be labeled as “green” and “safe” from a chemical point of view, they showed a clear stimulating effect of *R. subcapitata* growth. This effect was not always observed when single components were assessed at the same concentration as in the relative whole NADES, suggesting a putative synergistic effect for most of the substances while in the mixture. In particular, the NADESs with the most accentuate synergistic behavior were those containing betaine and proline, followed by those containing choline acetate and, less effectively, choline bitartrate. From observing different HBA/HBD combinations, results suggest that the hypothetical synergistic effect may be mainly linked to HBA contribution. Considering that, it appears quite clear that to label NADESs as “green” or “eco-friendly”, a deeper investigation on both molecular behavior in solutions and interactions at several organization levels, from molecules to ecosystem, is necessary in order to prevent unpredictable negative effects on the environment. 

## Data Availability

The original contributions presented in this study are included in the article. Further inquiries can be directed to the corresponding authors.

## Supplementary Materials

The following supporting information can be downloaded at: https://www.mdpi.com/article/10.3390/molecules31020262/s1, Table S1: Tukey’s multiple comparisons test data.

## Abbreviations

The following abbreviations are used in this manuscript:

ANOVA	Analysis of Variance
BBM	Bold Basal Medium
Bet	Betaine
CA	Citric Acid
CBT	Closed Bottle Test
ChA	Choline Acetate
ChCl	Choline Chloride
ChTA	Choline Tartarate (Bitartrate)
DES	Deep Eutectic Solvents
DGA	Diglycolic Acid
DMF	N,N-Dimethylformamide
EG	Ethylene Glycol
GA	Glycolic Acid
HBA	Hydrogen Bond Acceptor
HBD	Hydrogen Bond Donor
Im	Imidazole
ILs	Ionic Liquids
LA	Levulinic Acid
L-LacA	L-Lactic Acid
L-Pro	L-Proline
MA	Malic Acid
NADESs	Natural Deep Eutectic Solvents
OECD	Organisation for Economic Co-operation and Development
ThOD	Theoretical Oxygen Demand
WWTP	Wastewater Treatment Plant

## Appendix A

**Table A1:** NADESs composition and molar ratio.

NADES	HBA	HBD	Molar Ratio
1.Bet:EG 2.Bet:CA:water 3.Bet:Gly 4.Bet:LA 5.Bet:L-LacA		L-lactic acid	1:4 2:1:6 1:2 1:2 1:1
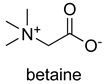
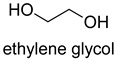
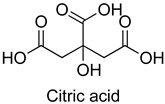
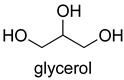
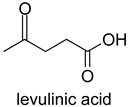
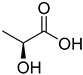
6. L-Pro:Gly 7.L-Pro: L-LacA 8.L-Pro:LA 9.ChTA:CA 10.L-Pro:MA	L-proline L-proline L-proline Choline bitartrate L-proline	L-lactic acid D,L-malic acid	1:2.5 1:1 1:2 1:1 1:1
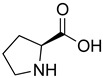
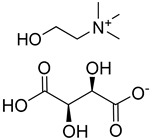
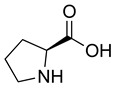
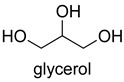
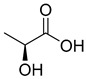
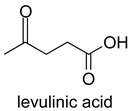
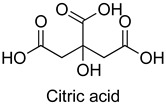
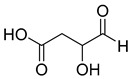
11.ChA:Im 12.ChA:LA 13.ChA:GA 14.ChA:DGA 15.ChA:CA		Imidazole	1:1 1:1 1.1 1:1 1:1
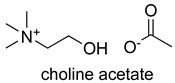
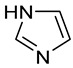
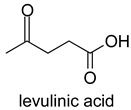
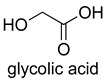
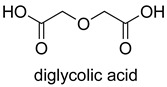
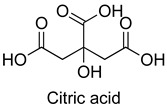

**Table A2:** NADESs and their single components, with the relative brutto-formula, molar mass, concentration for biodegradability test, and biodegradation achieved by CBT 301D (28 days).

NADES No.	HBA:HBD (Molar Ratio)	Structural Formula	Molar Mass (g/mol)	Test Substance (mg/L)	Biodegradation (28 days)
1	Bet:EG (1:4)	C_13_H_35_NO_10_	365.42	3.57	76%
2	Bet:CA:water (2:1:6)	C_16_H_42_N_2_O_17_	371.86	5.06	70%
3	Bet:Gly (1:2)	C_11_H_27_NO_8_	301.34	3.62	67%
4	Bet:LA (1:2)	C_15_H_27_NO_8_	229.66	4.78	68%
5	Bet:L-LacA (1:1)	C_8_H_17_NO_5_	263.759	3.60	70%
6	L-Pro:Gly (1:2.5)	C_13_H_31_NO_10_	387.895	3.76	74%
7	L-Pro + L-LacA (1:1)	C_8_H_15_NO_5_	205.210	3.77	65%
8	L-Pro:LA (1:2)	C_10_H_21_NO_4_	347.364	3.29	69%
9	ChTA:CA (1:1)	C_12_H_28_N_2_O_6_	445.374	4.97	60%
10	L-Pro:MA (1:1)	C_9_H_15_NO_7_	249.219	4.58	61%
11	ChA:Im (1:1)	C_10_H_21_N_3_O_3_	231.30	3.14	67%
12	ChA:LA (1:1)	C_12_H_25_NO_6_	279.33	3.01	81%
13	ChA:GA (1:1)	C_9_H_21_NO_6_	239.27	3.56	80%
14	ChA:DGA (1:1)	C_11_H_23_NO_8_	297.30	3.87	77%
15	ChA:CA (1:1)	C_13_H_25_NO_10_	373.36	4.32	66%
Single NADES components					
Betaine		C_5_H_11_NO_2_	117.148	3.05	76%
Ethylene glycol		C_2_H_6_O_2_	62.068	3.88	65%
Citric Acid		C_6_H_8_O_7_	192.123	6.67	76%
Glycerol		C_3_H_8_O_3_	92.094	4.11	75%
Levulinic Acid		C_5_H_8_O_3_	116.116	3.30	72%
L-Lactic Acid		C_3_H_6_O_3_	90.078	4.69	65%
L-Proline		C_5_H_9_NO_2_	115.132	3.27	72%
D,L-Malic Acid		C_4_H_6_O_5_	134.087	6.98	73%
Choline Acetate		C_7_H_17_NO_3_	163.21	2.83	75%
Imidazole		C_3_H_4_N_2_	68.077	4.25	23%
Glycolic Acid		C_2_H_4_O_3_	76.05	7.92	79%
Diglycolic Acid		C_4_H_6_O_5_	134.09	6.98	76%
Choline Bitartrate		C_9_H_19_NO_7_	253.251	4.17	66%
